# Pullulanase: Role in Starch Hydrolysis and Potential Industrial Applications

**DOI:** 10.1155/2012/921362

**Published:** 2012-09-06

**Authors:** Siew Ling Hii, Joo Shun Tan, Tau Chuan Ling, Arbakariya Bin Ariff

**Affiliations:** ^1^Department of Chemical Engineering, Faculty of Engineering and Science, Universiti Tunku Abdul Rahman, 53300 Kuala Lumpur, Malaysia; ^2^Institute of Bioscience, Universiti Putra Malaysia, 43400 Serdang, Selangor, Malaysia; ^3^Institute of Biological Sciences, Faculty of Science, University of Malaya, 50603 Kuala Lumpur, Malaysia; ^4^Department of Bioprocess Technology, Faculty of Biotechnology and Biomolecular Sciences, Universiti Putra Malaysia, 43400 Serdang, Selangor, Malaysia

## Abstract

The use of pullulanase (EC 3.2.1.41) has recently been the subject of increased applications in starch-based industries especially those aimed for glucose production. Pullulanase, an important debranching enzyme, has been widely utilised to hydrolyse the **α**-1,6 glucosidic linkages in starch, amylopectin, pullulan, and related oligosaccharides, which enables a complete and efficient conversion of the branched polysaccharides into small fermentable sugars during saccharification process. The industrial manufacturing of glucose involves two successive enzymatic steps: liquefaction, carried out after gelatinisation by the action of **α**-amylase; saccharification, which results in further transformation of maltodextrins into glucose. During saccharification process, pullulanase has been used to increase the final glucose concentration with reduced amount of glucoamylase. Therefore, the reversion reaction that involves resynthesis of saccharides from glucose molecules is prevented. To date, five groups of pullulanase enzymes have been reported, that is, (i) pullulanase type I, (ii) amylopullulanase, (iii) neopullulanase, (iv) isopullulanase, and (v) pullulan hydrolase type III. The current paper extensively reviews each category of pullulanase, properties of pullulanase, merits of applying pullulanase during starch bioprocessing, current genetic engineering works related to pullulanase genes, and possible industrial applications of pullulanase.

## 1. Introduction

Starch is a major industrial raw material and is chemically and/or enzymatically processed into variety of products for subsequent use in various industries, ranging from food (especially high-fructose and glucose syrups) to washing detergent industries [1–3]. Starch is, after cellulose, one of the most abundant heterogeneous polysaccharide produced by plants in the form of water insoluble granules. It is a polymeric carbohydrate, composed of C, H, and O atoms in the ratio of 6 : 10 : 5, (C_6_H_10_O_5_)_*n*_. Molecules of starch are made of hundreds or thousands of glucose, corresponding to values of *n* that range from 50 to several thousands. Glucose units are linked to one another through C_1_ oxygen as glucosidic bond. Glucosidic bonds are stable under alkaline conditions while treatment of starch with acids or certain enzymes breaks the polymer into its constituent glucose molecules. The end unit of the polymeric chain has a latent aldehyde group and is known as the reducing end group.

Most starches are mixture of two polymers with high molecular weight: (i) a linear chain molecule—amylose, and (ii) a branch polymer of glucose—amylopectin. Starches of different origins have different amylose and amylopectin ratios which differ significantly in many physical properties [4, 5]. In addition, the ratio of amylose to amylopectin in starch varies considerably depending on the origin, plant species, variety within plants, plants organs, age of organ, and growth conditions. This results in various crystalline organization of starch in the granules and henceforth different sensitivities of starch to enzymatic hydrolysis [6].

**Figure 1:**
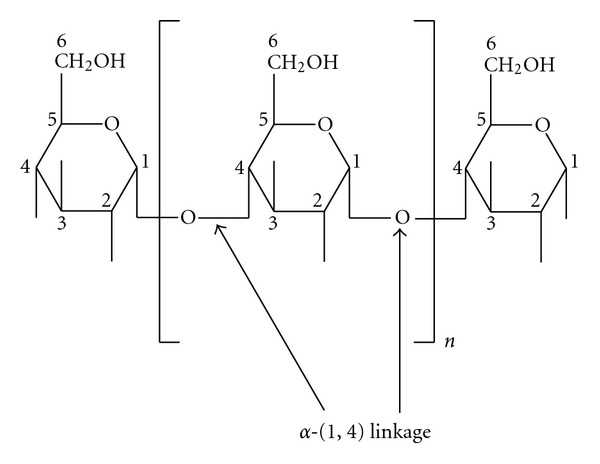
Section of the amylose molecule showing the repeating anhydroglucose unit (modified from [8]).

Amylose is the constituent of starch with anhydroglucose units linked by *α*-D-1,4 glucosidic bonds to form linear chains (Figure 1). A trace amount of branching is also present in the amylose molecule, although the side chains through branches are shorter in amylose than those in amylopectin [5]. The level of amylose and its molecular weight varies with types of starch. In addition, degree of polymerization (DP) of amylose covers a wide range, depending upon the starch source and origin. Aqueous solutions of amylose are very unstable due to intermolecular attraction and association of neighboring amylose molecules, which leads to increase in viscosity [7]. Retrogradation and precipitation of amylose particles may occur under certain specific conditions. 

**Figure 2:**
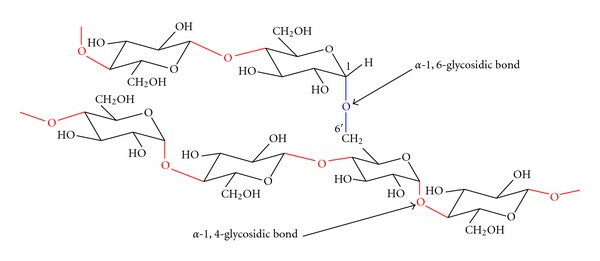
Section of the amylopectin molecule showing the *α*-1, 4 and *α*-1,6 chain linkages in starch (modified from [8]).

**Figure 3:**
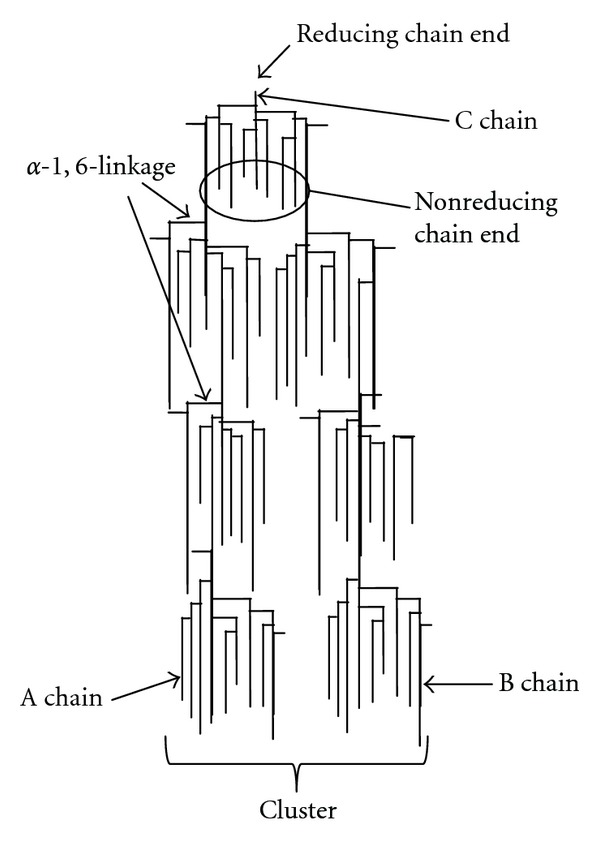
A diagram showing how the side-branching chains are clustered together within the amylopectin molecule (modified from [7]).

Amylopectin is one of the largest molecules in nature with an average DP of about 2 million. The molecular weight of amylopectin is about 1000 times as high as the molecular weight of amylose [8]. In addition to *α*-D-1,4 bonds which are present in amylose and the linear segments of amylopectin, amylopectin molecule has *α*-D-1,6 bonds which occur every 20 to 30 anhydroglucose units (Figure 2). Glucose units with an *α*-1,6 glucosidic linkages are the branching points which cause interlinkages of glucose residue that give rise to a ramified of bush-like structure of amylopectin molecule [9]. The branches in the amylopectin are not randomly organized but are clustered of 7 nm to 10 nm intervals (Figure 3), which are the universal features in structure of starch, regardless the source of botanical plant [10]. The branches themselves form an organized 3D structure: A-chains are not substituted at the C_6_ positions and inner B-chains are *α*-(1,6)-branches at one point or several points. The branching points make up about 5% glucose unit in amylopectin [7]. Generally, most cereal starches possess A-chains pattern, while cereal starches rich in amylase, tuber such as potato and rhizome such as canna yield the B-chains pattern, where C-chains patterns are normally found in legume starches [10].

Aqueous solutions of amylopectin are characterised by high viscosity, clarity, stability, and resistance to gelling. Amylopectin binds weakly with iodine and the complex typically gives a red/brown color. The level of amylopectin varies with starch types. The majority of starches used in the manufacture of glucose syrups contain 75% to 85% of amylopectin [11] and in most plants, ranging from 60% up to 90%, and reached about 100% in waxy cultivars of rice, maize, sorghum, barley, pea, and potato [5].

## 2. Starch-Converting Enzymes

**Figure 4:**
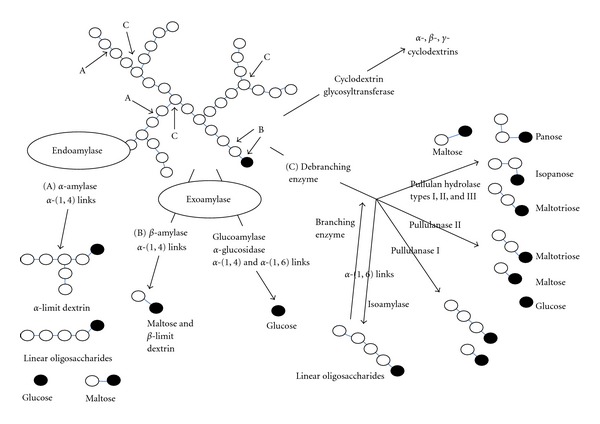
Schematic presentation of the action of amylases. Black circles indicate reducing sugars (modified from [17]).

The starch polymer, because of its complex structure, requires a combination of enzymes (endoamylases, and exoamylases) for the depolymerization of starch into oligosaccharides and smaller sugars, or to transform starch by transferring oligoglucosidic linkages and residues by the creation of new bonds (debranching enzymes and glycosyl-transferases) [3, 12, 13]. The enzymes commonly used for starch processing are generically classified as amylases [12, 14]. The process of enzymatic starch conversion is displayed in Figure 4. Basically, there are four types of starch-converting enzymes: (i) endoamylases; (ii) exoamylases; (iii) debranching enzymes; (iv) transferases.

### 2.1. Endoamylases

Endo-acting enzymes or endoamylases are able to cleave *α*-1,4 glucosidic bonds present in the inner part (endo-) of the amylose or amylopectin chain. Enzyme *α*-amylase (EC 3.2.1.1) is a well-known endoamylase that hydrolyses *α*-1,4 linkages in the interior part of starch polymer in a random fashion, which leads to the formation of linear and branched oligosaccharides, or *α*-limit dextrins. These enzymes are found in a wide variety of microorganisms, belonging to *Bacteria *and *Archaea *[12, 15]. 

### 2.2. Exoamylases

Exoamylases such as glucoamylases (EC 3.2.1.3) and *α*-glucosidases (EC 3.2.1.20) cleave both *α*-1,4 and *α*-1,6 bonds on the external glucose residues of amylose or amylopectin from the nonreducing end and thus produce only glucose (glucoamylase and *α*-glucosidases). On the other hand, *β*-amylases (EC 3.2.1.2) that cleave exclusively *α*-1,4 bonds produce maltose and *β*-limit dextrin [12, 13, 16, 17].

### 2.3. Transferases

Transferases is another group of starch-converting enzymes that cleave an *α*-1,4 glucosidic bond of the donor molecule and transfer part of the donor to a glucosidic acceptor with the formation of a new glucosidic bond. Enzymes such as amylomaltase (EC 2.4.1.25) and cyclodextrin glycosyltransferase (EC 2.4.1.19) form a new *α*-1,4 glucosidic bond while branching enzyme (EC 2.4.1.18) forms a new *α*-1, 6 glucosidic bond [3, 12]. The third group of starch-converting enzymes is the debranching enzymes and is described in details in the following section. 

## 3. Starch-Debranching Enzymes

Debranching enzymes catalyse the hydrolysis of *α*-1,6-glucosidic bonds in amylopectin and/or glycogen and related polymers. The affinity of debranching enzymes for the *α*-1,6-bond distinguishes these enzymes from other amylases which have primary affinity for *α*-1,4-glucosidic linkages. Debranching enzymes are classified into two major groups, that is, direct and indirect [19]. As shown in Table 1, these enzymes may be divided into three major groups according to their substrate specificity: (a) microbial pullulanases and plant R-enzymes (pullulan-6-glucanohydrolases), (b) isoamylases (glycogen-6-glucanohydrolases), and (c) amylo-1,6-glucosidases (dextrin 6-*α*-D-glucosidases) found in higher microorganisms [5, 14, 18, 19].

### 3.1. Indirect Debranching Enzyme

**Figure 5:**
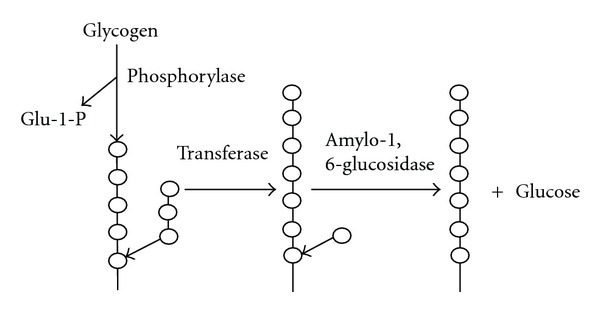
Action of amylo-1,6-glucosidase (modified from [18]).

The indirect debranching enzyme, that is, amylo-1,6-glucosidase, requires a modification of the substrate by another enzyme or enzymes prior to their debranching action [18, 19] and it is normally present in animal and yeast [5]. The amylo-1,6-glucosidase first requires the removal of an oligosaccharide by transglucosylase (4-*α*-glucanotransferase, EC 2.4.1.25), leaving a glucose residue bound to a tetrasaccharide through a 1,6-bond [5, 14, 18]. Amylo-1,6-glucosidase will only hydrolyse a 1,6-*α*-branch point if the side chain consists of a single glucose unit. The smallest known substrate that amylo-1,6-glucosidase can hydrolyze is the branched pentasaccharide 6^3^-*α*-glucosyl maltotetraose, which is hydrolyzed to glucose and maltotetraose (Figure 5). Amylo-1,6-glucosidases are not of industrial importance at present, so their properties and function are not described further in this paper.

### 3.2. Direct Debranching Enzymes—Pullulanase and Isoamylase

Direct-acting debranching enzymes which are present in plants and bacteria can directly hydrolyse *α*-1, 6-glucosidic bonds of unmodified substrate and classified into pullulanases, isoamylases, or R-enzymes on the basis of substrate specificity.

**Pullulanase**

**Figure 6:**
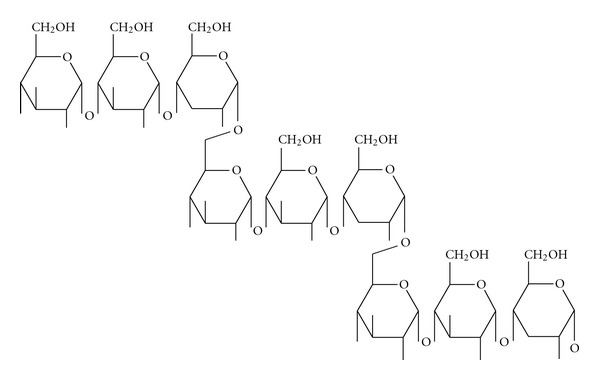
Generalized structure of pullulan from *Aureobasidium pullulans* strain CH-1 (modified from [2]).

Pullulanase with EC 3.2.1.41 or also known as *α*-dextrin 6-glucanohydrolase, pullulan 6-glucanohydrolase, limit dextrinase, and amylopectin 6-glucanohydrolase is derived from various microorganisms such as *Bacillus acidopullulyticus*, *Klebsiella planticola* [21], *Bacillus deramificans *[22], thermophilic *Bacillus* sp. AN-7 [23], *Bacillus cereus* FDTA-13 [24], and *Geobacillus stearothermophilus* [25]. Microbial pullulanase attracts more interest because of its specific action on *α*-1,6 linkages in pullulan, a linear *α*-glucan consisting essentially of maltotriosyl units connected by 1,6-*α*-bonds. The structure of pullulan produced by the fungus, *Aureobasidium pullulans*, is shown in Figure 6.

To date, five groups of pullulan-hydrolyzing enzyme have been reported in the literature and the details are summarised in Table 2. Enzymes hydrolysing pullulan are classified into groups based on the substrate specificities and reaction products. Pullulanases type I, which are able to hydrolyse efficiently the *α*-(1,6) glucosidic bonds in pullulan and branched polysaccharides, have been extensively studied [26–30]. Pullulanases type II, also called amylopullulanases, are prominent in starch processing industry due to the specific debranching capacity of hydrolysing either *α*-(1,6) or *α*-(1,4) glucosidic linkages. This enzyme debranch pullulan and gives maltotriose as final product and it also attacks *α*-(1,4) bonds in starch, amylose, and amylopectin [3, 12, 31]. Both pullulanases type I and type II attach *α*-1,6 glucosidic linkages in pullulan, producing maltotriose while these enzymes are unable to degrade cyclodextrin [32].

Very few reports are available on other pullulanases. Pullulan hydrolase type I (neopullulanase) and type II (isopullulanase) are only able to cleave *α*-1,4 glucosidic linkages in pullulan, releasing panose and isopanose, and are highly active on cyclodextrins [32–34]. The enzymes that degrade cyclodextrins faster than starch are sometimes designated cyclodextrinases (EC 3.2.1.54, cyclomaltodextrinase) [3, 34]. Interestingly, cyclomaltodextrinases are intracellular enzymes whereas most enzymes involved in starch conversion are extracellular [35]. These two types of pullulan-hydrolysing enzyme (neopullulanase and isopullulanase) have practically no action on starch [36]. As these enzymes can precisely recognise the structural differences between *α*-1,4 and *α*-1,6 glucosidic linkages, they are widely applied in structural analysis of oligo- and polysaccharides [31]. 

Unlike all pullulan-hydrolysing enzymes as described above, pullulan hydrolase type III detected by Niehaus et al. [37] has ability to attack *α*-(1,6) as well as *α*-(1, 4)-glucosidic linkages in pullulan leading to the formation of a mixture of maltotriose, panose, maltose, and glucose. The enzyme is also able to degrade starch, amylose, and amylopectin forming maltotriose and maltose as main products.

**Isoamylase**

Isoamylase (EC 3.2.1.68)(glycogen 6-glucanohydrolase) is the only known enzyme that debranches glycogen completely. This enzyme contains two subunits with a molecular weight of 45 000, pI of pH 4.4 and requires no metal cofactor. This enzyme is most active at pH ranging from pH 3 to pH 4 and has maximum temperature stability at 45°C to 55°C [1].

### 3.3. Comparisons between Pullulanase and Isoamylase

The major difference between pullulanase and isoamylase is that pullulanases hydrolyse the *α*-1,6 glucosidic bond in pullulan and amylopectin, while isoamylase can only hydrolyse the *α*-1,6 bond in amylopectin and glycogen [12]. Pullulanase requires each of the two chains linked by an *α*-1,6- glucosidic bond contains at least two *α*-1,4-linked glucose units. Thus, the smallest substrate for pullulanase is the tetrasaccharide 6^2^-*α*-maltosylmaltose. In contrast, isoamylase prefers substrates with high-molecular-weight oligosaccharides and the smallest substrate meeting this requirement is the pentasaccharide 6^3^-*α*-maltosyl maltotriose such as glycogen from animal sources [38]. The fine structure of amylopectin is distinct from that of glycogen in animals and bacteria as glycogen is randomly branched. The branches are more numerous, and the chains are shorter as compared to amylopectin [39].

The debranching enzyme from yeast and mold is known as isoamylase, and the enzymes produced by bacteria such as *Aerobacter aerogenes *or *Klebsiella aerogenes *are called pullulanase [14]. A comparison of the action of *Klebsiella pneumoniae *pullulanase and *Pseudomonas amyloderamosa *isoamylase on various substrates is given in Table 3. Although differing in their specificity, both types of enzymes directly hydrolyse *α*-1,6 linkages in amylopectin, removing side branches of various lengths from the main polymer chain. The main chain and the side branches are then completely available for hydrolysis by other hydrolytic enzymes [10, 38].

During starch saccharification processes, formation of *α*-limit and *β*-limit dextrins is the common phenomenon when amylopectin is treated with either *α*-amylase or *β*-amylase, respectively. The concentrations of final glucose obtained in the saccharification are greatly reduced due to the present of these limit dextrins [38]. The bacterial pullulanases are able to hydrolyse amylopectin and its *β*-limit dextrins and attack the partially degraded polymer (Table 3). Therefore, pullulanases from either *Klebsiella planticola *or *Bacillus acidopullulyticus *are more commonly used than isoamylase (e.g., from *Pseudomonas amylodermosa*), mainly because of their greater temperature stability and more *β*-amylase-compatible pH range [10]. Another disadvantage of using isoamylase during saccharification process is the inability to hydrolyse 2- and 3-glucose unit side chains in *β*-limit and *α*-limit dextrins. Thus, simultaneous action of *β*-amylase and isoamylase cannot quantitatively converts amylopectin to maltose [19]. The *β*-amylases are also found to be a potential inhibitor for the isoamylases activity [22]. Pullulanase, on the other hand, is generally used in combination with amyloglucosidase, *α*-amylase, and *β*-amylase. Furthermore, the presence of maltotriose or maltotetraose in the fluid competitively inhibits isoamylase action [21].

The most established advantage of using pullulanase instead of isoamylase during starch saccharification process is that the time of addition of isoamylase to the hydrolysis system is very critical. For example, when isoamylase is used before amyloglucosidase, the amylopectin fraction is depolymerised too rapidly and therefore is highly susceptible to retrogradation [44]. 

### 3.4. Advantages of Using Pullulanase in Starch Saccharification Processes

**Figure 7:**
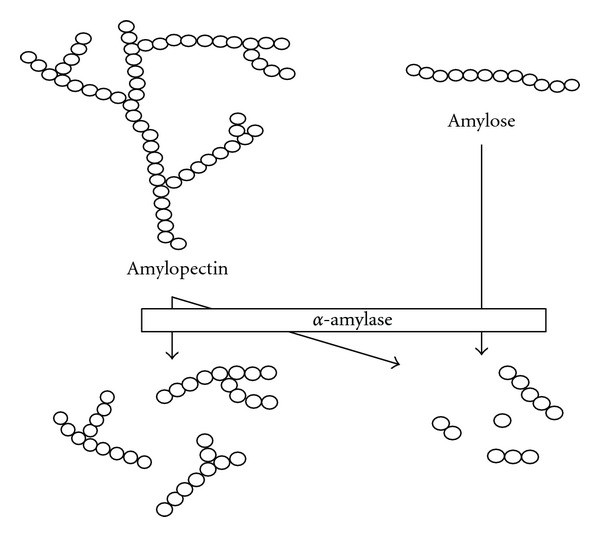
Partial hydrolysis of starch by *α*-amylase (modified from [11]).

The majority of starches which are of industrial importance contain approximately 80% amylopectin (Table 4). The branch points occur on average every 20 to 25 D-glucose units, so that amylopectin contains 4% to 5% of *α*-1,6 glucosidic linkages [11]. During starch conversion process, starch is first gelatinised and solubilised at high temperatures and the long-chain molecules broken down into smaller units (maltodextrins) which can be either in the form of branch or linear (Figure 7).

The *α*-1, 6-glucosidic linkages present in starch molecules act as a kind of barrier to the action of various starch hydrolysing enzymes during the saccharification process that followed after gelatinisation process. When starch is subjected to hydrolysis by *α*-amylases, this endo-acting enzyme is able to bypass the branch points, but in general is not capable of hydrolyzing the *α*-1,6 glucosidic linkage. As a result, the amylopectin fraction is only partially degraded [11, 18]. The branch points containing *α*-1,6 glucosidic linkages are resistant to attack and their presence also imposes a certain degree of resistance on neighbouring *α*-1,4 linkages. Thus, the prolonged action of *α*-amylase on amylopectin results in the formation of “*α*-limit dextrins," which are not susceptible to further hydrolysis by the *α*-amylase (Figure 7). Similarly, when amylopectin is treated with *β*-amylase, hydrolysis stops as a 1,6-*α* branch point is approached, resulting in the formation of *β*-limit dextrins [18].

**Figure 8:**
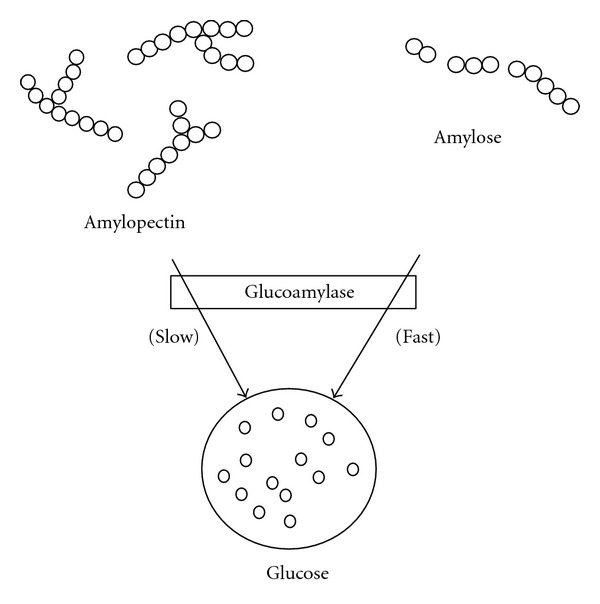
Action of glucoamylase on liquefied starch (modified from [11]).

During saccharification, partially hydrolysed amylose and amylopectin molecules are depolymerised by the action of glucoamylase which removes glucose units in a stepwise manner from the nonreducing chain ends [11, 45]. The rate of hydrolysis by glucoamylase depends on the particular linkage, size of molecule, and the order in which *α*-1,4 and *α*-1,6 linkages are arranged [45]. Glucoamylase hydrolyses the *α*-1,4 links very efficiently, and at a much slower rate for *α*-1,6 links. For example, the rates of hydrolysing 1,4-*α*, 1,6-*α* and 1,3-*α*-links in tetrasaccharides are in the proportion of 300 : 6 : 1 [11, 46]. As shown in Figure 8, glucoamylases slowly hydrolyse 1,6-*α*-glucosidic linkages in amylopectin and partially hydrolyse amylopectin, but the action of this maltogenic exoamylases ceases or stops as a branch point is approached [11]. 

Glucoamylase also catalyses the reverse reaction (reversion), in which dextrose molecules are combined to form maltose and isomaltose [11, 38, 47]. When *α*-amylase and glucoamylase are used in sequence to saccharify sago starch, the isomaltose is produced [48]. However, treatment with a mixture of glucoamylase and pullulanase during the saccharification of sago starch resulted in the production of glucose but no isomaltose. The reversion of dextrose specifically involves the condensation of a *β*-anomer of D-glucopyranose with either an *α*- or a *β*-D-glucose molecule in the presence of glucoamylase, as shown below:

(1) $$\begin{array}{c} D\text{-glucose}+\beta \text{-}D\text{-glucose}\to \text{Dissacharide}+{\text{H}}_{2}\text{O}. \end{array}$$

**Figure 9:**
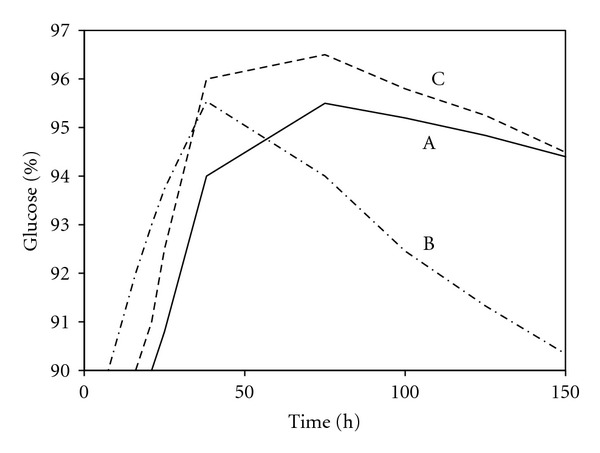
The percentage (%) of glucose formed from maltodextrin using various enzyme solutions. Symbols: A, 200 U/kg *Aspergillus niger *glucoamylase; B, 400 U/kg *Aspergillus niger *glucoamylase; C, 200 U/kg *Aspergillus niger *glucoamylase plus 200 U/kg *Bacillus acidopullulyticus *pullulanase (modified from [20]).

**Figure 10:**
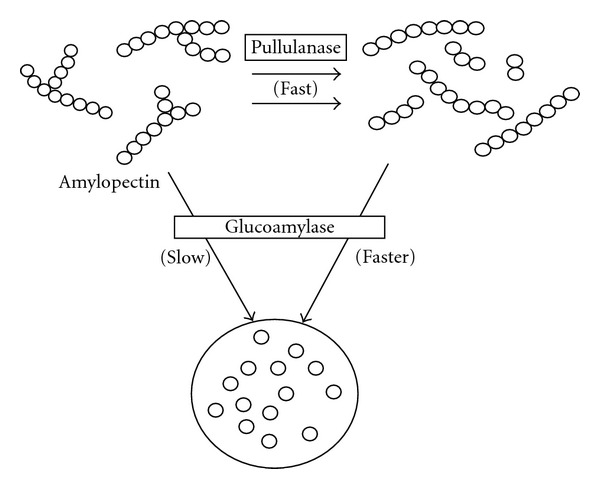
Effect of pullulanase during saccharification (modified from [11]).

Isomaltose cannot be enzymatically hydrolysed into smaller molecule [22]. In industry, isomaltose is byproducts that decrease overall glucose yield and are unacceptable in the final high-fructose syrup used as sweetener [49].  Figure 9 shows the incubation of maltodextrin with high loading of glucoamylase which results in reduction of final glucose concentration due to continuous formation of isomaltose [12, 46]. Hence, minimising this reaction is important for increment of the final glucose yield in industrial process [45]. The efficiency of saccharification reaction could be improved by incorporating a specific amylopectin-debranching enzyme in the system. If a debranching enzyme, such as pullulanase and glucoamylase, is simultaneously used during saccharification, the pullulanase would specifically hydrolyse the branch points in the amylopectin residues, followed by the hydrolysis of linear 1,4-*α*-glucosidic linkages by glucoamylase (Figure 10). As a result, the maximum dextrose levels that could be achieved are higher (Figure 9) [20, 38].

The use of a debranching enzyme would increase the rate of overall saccharification process and reduce the total amount of glucoamylase that is required for complete conversion process. The practical advantage of using pullulanase together with glucoamylase is that less glucoamylase activity is needed. Reduction in the use of up to 60% glucoamylase has been reported [11, 50]. This does not in itself give any cost advantage. However, less glucoamylase is used and fewer branched oligosaccharides accumulated toward the end of the saccharification and this is the point at which isomaltose production becomes significant [20].

**Figure 11:**
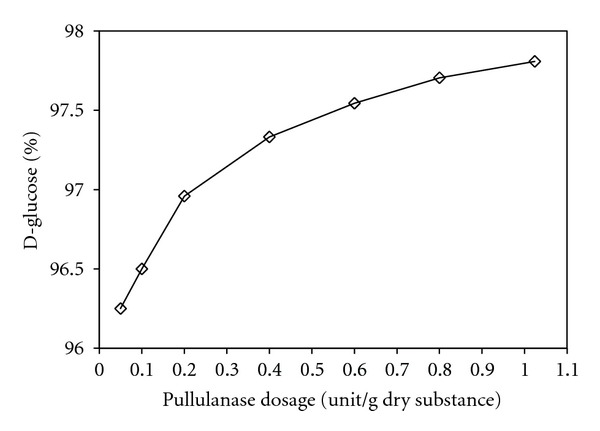
The effect of pullulanase activity on maximum D-glucose concentration (modified from [11]).

The effect of pullulanase activity on the increment of D-glucose production is illustrated in Figure 11. In the standard saccharification process, without pullulanase, the maximum D-glucose that could be obtained under the given conditions is about 96%. By using pullulanase, substantially lower glucoamylase dosage is possible to increase the D-glucose level by about 2%. In addition, using pullulanase together with *β*-amylase in starch saccharification process, maltose yield could be increased by about 20 to 25% [11, 50]. By using pullulanase in the system, increase in crystallization yield could also be achieved for dextrose production. In order to obtain the targeted fructose concentration, pullulanase has been employed in fructose syrup production process, which in turn reduced the isomerization costs. For example, polysaccharide content in the final product can be reduced by up to about 50% in the production of 55% fructose syrup [11].

**Figure 12:**
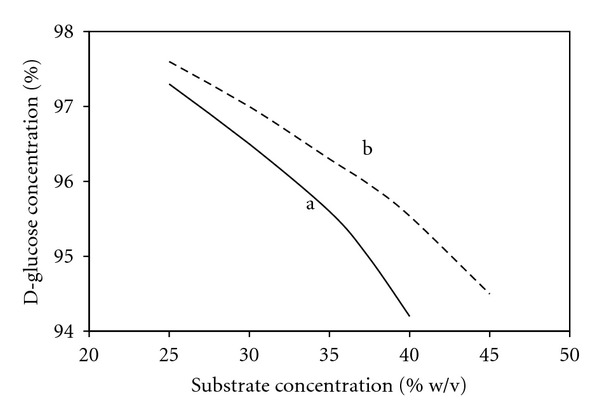
The effect of substrate concentration on maximum D-glucose concentration. Symbols: a, without pullulanase; b, with pullulanase (modified from [11, 18]).

The effect of pullulanase on the amount of substrate that can be treated to produce maximum D-glucose is shown in Figure 12. Saccharification may be carried out at higher substrate concentrations (30% to 40%) than in the normal process (25% to 30%) if a combination of a debranching enzyme and glucoamylase is used [20, 50]. Furthermore, the extra cost of using pullulanase is recouped by saving the cost of evaporation and glucoamylase. For high-fructose syrups production, cost of further processing could also be reduced [20].

**Figure 13:**
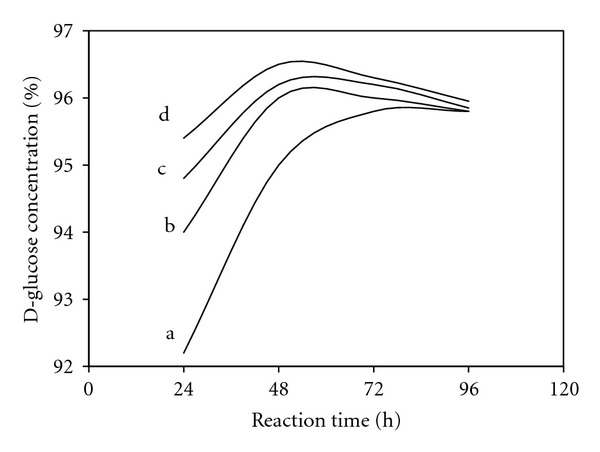
The effect of pullulanase dosage on reaction time. Symbols: a, without pullulanase addition; b, 0.04 unit of pullulanase/g dry solid; c, 0.08 unit of pullulanase/g dry solid; d, 0.16 unit of pullulanase/g dry solid (modified from [11]).

The capacity of syrup production plant could be increased by reducing the starch saccharification time. In a conventional process, saccharification time could be increased by using high glucoamylase dosage and the time could be reduced from 45 to 30 h by doubling the dosage. Unfortunately, the reversion reaction (isomaltose formation from D-glucose) is very significant, and its therefore difficult to stop the saccharification at, or close to, maximum D-glucose. If normal glucoamylase dosage with a combination of a debranching enzyme is used, the reaction time could be reduced without the problems of overconversion [11]. As illustrated in Figure 13, for process without the debranching enzyme, maximum D-glucose was obtained after about 80 h of reaction. When the pullulanase loading is increased, the same level of D-glucose can be attained at shorter incubation time (30 h).

### 3.5. Properties of Pullulanases

As mentioned earlier, enzymes which hydrolyse pullulan have been classified into five major groups. A number of pullulanases have been purified and characterised from different bacterial sources (Table 5). Pullulanase type I has been characterised from mesophilic bacteria such as *Aerobacter aerogenes* [69], *Bacillus acidopullulyticus *[11], *Klebsiella pneumonia *[70, 71], and *Streptomyces *sp. [52]. Moderate thermophilic gram-positive bacteria such as *Bacillus flavocaldarius *[51], *Bacillus thermoleovorans *[30], *Clostridium *sp. [72], and *Thermos caldophilus *[27] also have ability to secrete pullulanase type I while pullulanase type I from hyperthermophilic bacterium, *Fervidobacterium pennavorans, *has also been reported [28]. 

Unlike pullulanase type I, pullulanase type II is widely distributed among extreme thermophilic *Bacteria *and hyperthermophilic *Archaea* [43]. The most thermostable and thermoactive pullulanase type II reported to date was derived from the hyperthermophilic archaeon *Pyrococcus woesei *[26] and *Pyrococcus furiosus *[55, 73].The temperature optima for these thermoactive pullulanase were ranged from 85°C to 105°C and maintained its thermostability even in the absent of substrate and calcium ions [17]. The gene encoding the pullulanase of *Pyrococcus woesei *was cloned and expressed in *E. coli*, which was the first archaeal pullulanase that was cloned and expressed in a mesophilic host [26]. With the present of 5 mM Ca^2+^, the activity of pullulanase type II produced by *Pyrococcus furiosus *and *Thermococcus litoralis *was active and stable at temperature ranging from 130°C to 140°C [55]. Thermoactive pullulanase type II from *Desulfurococcus mucosus*, *Thermococcus celer, Thermococcus *strain TYS, and* Thermococcus *strain TY has also been reported [56]. However, this hyperthermophilic pullulanase type II has not been yet in commercial production due to low yield and limited activity at high starch concentrations (>30%) [74]. Several alkaline pullulanases type II, which were active at pH ranging from 8.5 to 12, have been isolated and characterized from *Bacillus *spp. [41, 59, 75, 76].

Neopullulanases have been reported from many types of microorganisms such as *Bacillus stearothermophilus *[42], *Bacteroides thetaiotaomicron *95-1 [57], and *Bacillus polymyxa *CECT 155 [58]. Neopullulanases hydrolyse pullulan to produce panose as the main product with the final molar ratio of panose, maltose, and glucose of 3 : 1 : 1 [77]. Neopullulanase is also closely related to cyclomaltodextrinases based on similarity of their specific reaction towards pullulan [34]. Acidic isopullulanases have been characterised from *Aspergillus niger *ATCC 9642 [36] and *Bacillus *sp. US 149 [31]. Recently, pullulan hydrolase type III has been isolated and characterised from hyperthermophilic archaeon, *Thermococcus aggregans *[37]. This is the only enzyme presently known that attacks *α*-1,4- as well as *α*-1,6-glucosidic linkages in pullulan and is active at above 100°C.

## 4. Genetic Engineering of Pullulanase 

Wild-type microorganisms normally produce low activity of pullulanase enzymes, which are not enough to meet the demand for biotechnological applications. Molecular cloning of the corresponding genes and their expression in heterologous hosts is one of the possible approaches to circumvent this problem. Large quantities of specific gene can be isolated in pure form by molecular cloning and the target DNA can be produced in large amounts under the control of the expression vector. Overexpression following the cloning step can significantly increase the enzyme yield by subcloning the target gene into a suitable expression vector. Another approach is to insert a signal sequence into the host bacteria for the transfer of ligated gene product to the periplasmic region [78]. Research related to pullulanase gene has been carried out since 1980s. Most of the pullulanase genes were isolated from *Bacillus *spp. (Table 6). In addition, pullulanase genes from *Anaerobranca gottschalkii*, *Desulfurococcus mucosus,* and *Klebsiella aerogenes* have also been reported [24, 29, 60]; *Escherichia coli* and *Bacillus subtilis* were the two commonly used host strains for the expression of pullulanase gene.

## 5. Industrial Applications of Pullulanase

### 5.1. Saccharification of Starch

Since 1960s, almost all processes to convert starch to glucose have changed to enzymatic hydrolysis from traditional acid method [79]. Today, much of the starch hydrolysates available in the market are enzyme-converted products of higher DE. Dextrose equivalent (DE) is a measure of reducing sugar on a dry basis, pure dextrose (glucose) being DE 100 and starch being close to DE 0. The enzymatic conversion of starch into glucose, maltose, and fructose for use as food sweeteners represents an important growth area in industrial enzyme usage. The primary application of pullulanase is in starch saccharification and the most important industrial application of pullulanase is in the production of high-glucose (30% to 50% glucose; 30% to 40% maltose) or high-maltose (30% to 50% maltose; 6% to 10% glucose) syrups [12, 80]. In the saccharification process, pullulanase is normally used in combination with glucoamylase or *β*-amylase [13, 20, 80]. 

### 5.2. Production of High-Maltose Corn Syrup 

High-maltose syrups have mild sweetness, low viscosity in solution, low hygroscopicity, and good thermal stability. It is well suited for numerous applications in food processing such as in the manufacturing of high-quality candy and ice cream [81]. In recent years, there has been an increasing interest for pure maltose in the pharmaceutical industry. Maltose may be used instead of D-glucose for intravenous feeding, where it can be administered at higher concentrations without elevating blood glucose levels [81]. Pure maltose may also be used as a starting material for the production of maltitol and crystalline maltitol [11].

### 5.3. Production of High-Fructose Corn Syrup 

High-glucose syrup is used as a carbon source in fermentation and feed for making high-fructose syrups and crystalline glucose [79, 82]. High-fructose syrup is produced by the treatment of high-dextrose syrup, especially high-glucose syrup (DE 95–96) with immobilized glucose isomerase [12]. High-fructose corn syrup (HFCS) is a high-quality and clean-tasting caloric sweetener. HFCS with very high DE value is required in the production of crystalline glucose. HFCS is also widely used in product formulation. It is cheaper and less caloric than sucrose but 1.2 to 1.8 times sweeter than sucrose on a dry weight basis [44]. Like high-maltose syrup, 90% HFCS is used in food for diabetics because it can be metabolized without insulin [83].

### 5.4. Starch Processing Industry

Pullulanase has also been used to prepare high-amylose starches, which have huge market demand [84]. High-amylose starches are of great interest and can be processed into “resistant starch” which has nutritional benefits [85]. Unlike normal starch, resistant starch is not digested in the small intestine but is fermented in the large intestine by gut bacteria, producing short-chain fatty acids such as butyrates that are beneficial for colon health. High-amylose starches are also used in adhesive products and in the production of corrugated board and paper [86].

### 5.5. Detergent

Some alkaline pullulanases have been used as effective additives in dishwashing and laundry detergents for the removal of starches under alkaline conditions [76, 87]. The effectiveness of these alkaline debranching enzymes can be enhanced in washwater when the amylopullulanase is used in combination with alkaline *α*-amylase because one single amylopullulanase can catalyze both debranching (*α*-1,6 hydrolytic) and liquefying (*α*-1,4 hydrolytic) reactions [41].

### 5.6. Production of Cyclodextrins (CDs)

Pullulanase has also been used to enhance the yield of cyclodextrins by the reaction of CGTase with gelatinized starches and maltodextrin syrups in the presence of cyclodextrins (CDs) complexing agents [88]. CDs have a wide range of applications in complexing materials in foods, pharmaceuticals, plastics, and agricultural products as emulsifiers, antioxidants, and stabilizing agents [89]. 

### 5.7. Others

Pullulanase also finds minor application in the manufacturing of low-calorie beer [79] and in baking industry as the antistaling agent to improve texture, volume, and flavor of bakery products [12]. It is also possible to use pullulanase as a dental plaque control agent [90].

## 6. Conclusions

Pullulanase capable of hydrolysing *α*-1,6 linkages of polymer is widely used in saccharification process for production of various useful materials such as maltose, amylose, and glucose, by debranching starch with and without *α*-amylase, *β*-amylase, or glucoamylase, respectively. Clearly, pullulanase could facilitate a major change in the current strategy for starch processing and hence, there is a strong demand for this enzyme around the world with a growing presence in the industries related to its usage. 

## Figures and Tables

**Table 1:** Carbohydrate structure requirements for hydrolysis of 1,6-bonds by debranching enzymes. Symbols: ↓: cleavage by enzyme mentioned; *∅*: reducing end.

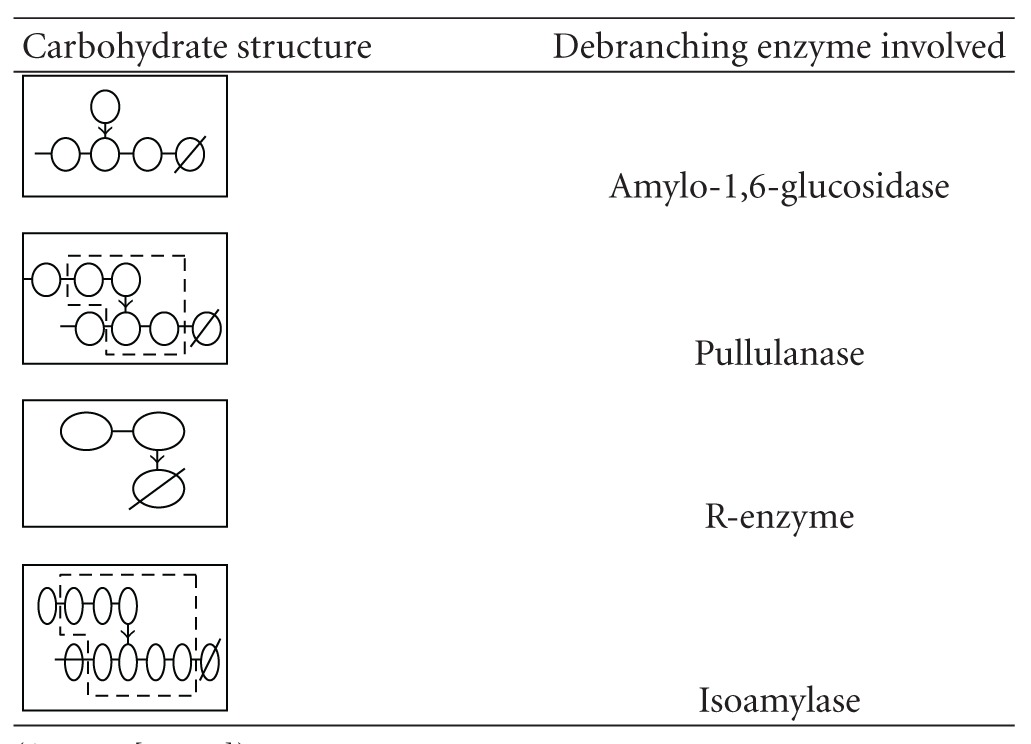

(Source: [18, 19]).

**Table 2:** Reaction specificities of pullulan-degrading enzymes.

Enzyme	EC number	Bonds processed	Preferred substrate	End products	References
Pullulanase type I	3.2.1.41	*α*-(1,6)	Oligo- and polysaccharides, Pullulan	Trimer (maltotriose)	[17, 27]

Pullulanase type II (amylopullulanase)		*α*-(1,6)	Pullulan,	Trimer (maltotriose)	
	3.2.1.41	*α*-(1,4)	Poly- and oligosaccharide (starch)	Mixture of glucose, maltose, and maltotriose	[31, 32, 40]

Pullulan hydrolase type I (neopullulanase)	3.2.1.135	*α*-(1,4)	Pullulan	Panose	[41–43]

Pullulan hydrolase type II (isopullulanase)	3.2.1.57	*α*-(1,4)	Pullulan	Isopanose	[12]

Pullulan hydrolase type III		*α*-(1,4) and *α*-(1,6)	Pullulan	Mixture of panose, maltose, and maltotriose	
	3.2.1.—		Starch, amylose, and amylopectin	Maltotriose and maltose	[37]

**Table 3:** Action of pullulanase and isoamylase on various substrates.

Substrate	Relative rate of hydrolysis	
	Isoamylase	Pullulanase
Pullulan	Very low	100
Pure amylopectin	100	15
Glycogen (oyster)	124	1
Rabbit liver	111	0.6

**Table 4:** Properties and composition of starch granules.

Starch	Type	Size range (diameter *μ*m)	Shape	Amylose (%)	Amylopectin (%)
Sago	Pith	5–65	Oval, truncated	27	73
Corn	Cereal	3–26	Round, polygonal	28	72
Potato	Tuber	5–100	Oval, spherical	21	79
Wheat	Cereal	2–35	Round, reticular	28	72
Tapioca	Root	4–35	Oval, truncated	17	83
Rice	Cereal	3–8	Polygonal, angular	17	83

(Source: [4, 5]).

**Table 5:** Physicochemical properties of selected pullulan-hydrolyzing enzymes from various microorganisms.

Organism	*M*_*r*_^a^ (kDa)	pI	Optimal pH	Optimal temperature (°C)	Reference
Type I pullulanase					
*Bacillus flavocaldarius *KP 1228	55	n.d.^b^	7.0	75–80	[51]
*Streptomyces *sp. No. 27	n.d.	n.d.	4–7	55–60	[52]
*Thermus caldophilus *GK-24	65	6.1	5.5	75	[27]
*Fervidobacterium pennavorans* Ven 5	240	n.d.	6.0	85	[28]
*Bacillus thermoleovorans* US 105 (expressed in *E. coli*)	n.d.	n.d.	5.6–6.0	75	[30]
Type II pullulanase					
*Thermoanaerobacter *strain B6A	450	4.5	5.0	75	[53]
*Clostridium thermosulfurigenes *EM 1	102	n.d.	5.5–6.0	60–65	[54]
*Pyrococcus furiosus *	110	n.d.	5.5	98	[55]
*Thermococcus litoralis *	119	n.d.	5.5	98	[55]
*Desulfurococcus mucosus *	n.d.	n.d.	5.0	100	[56]
*Thermococcus celer *	n.d.	n.d.	5.5	90	[56]
*Thermococcus *strain TY	n.d.	n.d.	6.5	100	[56]
*Pyrococcus woesei *	90	n.d.	6.0	100	[26]
*Thermococcus hydrothermalis *	110–128	n.d.	5.5	95	[14]
*Desulfurococcus mucosus *expressed in *Bacillus subtilis *	66	n.d.	5.0	85	[32]
Neopullulanases					
*Bacillus stearothermophilus *expressed in *B. subtilis *	62	n.d.	6.0	60–65	[42]
*Bacillus stearothermophilus* TRS 40	62	n.d.	6.0	60–65	[42]
*Bacteroides thetaiotaomicron *95-1	70	n.d.	6.5	37	[57]
*Bacillus polymyxa *CECT 155	n.d.	n.d.	6.0	50	[58]
Isopullulanases					
*Aspergillus niger *ATCC 9642	69–71	n.d.	3.5	40	[36]
*Bacillus *sp. US 149	200	n.d.	5.0	60	[31]
Alkaline amylopullulanase					
*Bacillus *sp. KSM-1876	120	5.2	10–10.5	50	[59]
*Bacillus *sp. KSM 1378	210	4.8	9.5	50	[41]
Pullulan hydrolase type III					
*Thermococcus aggregans *expressed in *E. coli *	n.d.	n.d.	6.5	95	[37]

^a^Molecular mass; ^b^not detected by the researchers.

**Table 6:** Genetic engineering of pullulanase gene.

Source	Expression strain	Length (a.a)*	Vector	References
*Anaerobranca gottschalkii*	*E. coli* BL21 (DE3)	865	pET21a	[60]
*Bacillus thermoleovorans *US105	*E. coli* DH5*α*	718	pEBM2	[30]
*Bacillus stearothermophilus *TS-23	*E. coli* XL1-Blue MRF	2018	pAP/StuI	[61]
*Bacillus stearothermophilus strain* TRS-128	*B. subtilis* NA-1	N.A.^#^	pTB522	[62]
*Klebsiella pneumonia*	*E. coli *K-12	N.A.^#^	pACYC184	[63]
*Thermus *IM6501	*E. coli*	718	p6xHis119	[64]
*Desulfurococcus mucosus*	*B. subtilis clone *JA803	686	JA803	[32]
*Fervidobacterium pennivorans *Ven5	*E. coli* FD748	849	pSE420	[29]
*Thermococcus hydrothermalis*	*E. coli *JM109 (DE)	1339	pAPUΔ2	[65]
*Thermotoga maritima*	*E. coli* JM83	840	pTPU1	[66]
*Caldicellulosiruptor saccharolyticus*	*E. coli *Q359	826	pNZ1038	[67]
*Bacillus *sp. XAL601 II	*E. coli* JM109	2032	pUC18	[68]

*a.a: amino acid.

^#^N.A.: not available.
